# How the Tower Air Traffic Controller Workload Influences the Capacity in a Complex Three-Runway Airport

**DOI:** 10.3390/ijerph18062807

**Published:** 2021-03-10

**Authors:** Paola Di Mascio, Riccardo Carrara, Luca Frasacco, Eleonora Luciano, Andrea Ponziani, Laura Moretti

**Affiliations:** 1 Department of Civil, Constructional and Environmental Engineering, Sapienza University of Rome, 00184 Rome, Italy; paola.dimascio@uniroma1.it (P.D.M.); carrara.1754371@studenti.uniroma1.it (R.C.); 2 ENAV Ente Nazionale Assistenza al Volo-Italian Air Navigation Service Provider Via Salaria, 00138 Rome, Italy; luca.frasacco@enav.it (L.F.); eleonora.luciano@enav.it (E.L.); andrea.ponziani@enav.it (A.P.)

**Keywords:** airport capacity, fast time simulation, aircraft delay simulation models, pareto frontiers, workload, tower air traffic controller, air traffic controller’s capacity

## Abstract

Air traffic controllers aim to optimize airport capacity, that is to increase the number of aircraft movements per hour maintaining a limited delay. There are several definitions of capacity, which depend on the considered airport element. This study focused on the development of a method that allows evaluating the impact of tower air traffic controllers’ workload on airport capacity. It adapts a model for the workload of sector controllers designed by Eurocontrol to tower controllers and tests it on a heavily busy international airport. In order to collect controllers’ working times, a campaign of data collection has been carried out from the radio frequency occupation. The results allowed us to extrapolate the hourly percentage of work of the various tower controllers using a fast-time simulation software. By imposing an hourly working threshold on tower air traffic controllers, it was possible to obtain a maximum number of manageable aircraft, which was compared with the airside capacity of the airport. The results show that the maximum traffic manageable from the airside would produce unacceptable workload for tower controllers, highlighting the link between airport capacity and the human component.

## 1. Introduction

Worldwide annual air passenger traffic exceeded 8.2 billion passengers in 2017 and it is expected to double by 2034 with a 4.3% annual growth rate [1]. Therefore, it will reach 20.9 billion passengers by 2040 [1]. In order to meet this rising traffic demand, it is necessary to adapt airport infrastructures and increase their efficiency. The variable used to quantify the performance of an airport is its capacity (i.e., the number of movements that can be dealt per unit time with a given infrastructure, by adhering to the reference regulation and taking a service quality level into account) [2].

The airport management body should analyze the current condition in order to identify when and where inefficiencies or risks occur. Before making any choice that involves a change in the airport infrastructure or/and procedures, the competent management body should simulate and evaluate different scenarios based on the potential performance. The verification of airport capacity needs when [3]:

(1) The current traffic demand is causing delays in good or bad weather conditions;
(2) An increase in demand for movements is expected;
(3) An adaptation of the airport is necessary due to an increase in safety standards or a change in the monitoring of airport services.

In the literature, several definitions of capacity are available: they depend on the authoritative entity, the time period taken as reference, the purpose of the study, and the infrastructural element of the capacitive study [3,4,5,6,7]. The common denominator of all these definitions is that they focus only on the infrastructural and procedural elements: the elements that influence capacity are the configuration of the runways and taxiways, the number and configuration of parking areas and gates, the presence and positioning of waiting areas, radar instrumentation, traffic composition in wake turbulences and procedures [8]. However, other elements such as the economic situation, migration, meteorology and topography affect the airport capacity [9,10], and there has been research regarding what happens if the human component is taken into account [11]. The bottleneck, or one of the critical issues, of the overall airport system could be Tower Air Traffic Controllers (TATC). Indeed, TATC workload is one of the most important factors determining the maximum allowable operations [12], but it should stay within acceptable boundaries to avoid overload conditions [13,14] and prevent negative effects on system safety and public health in the area surrounding the airport [15,16]. The work of air traffic controllers (ATCs) implies a predominantly mental load because it is composed of information interpretation and decision-making tasks [17]. The mental workload differs from person to person and depends on [3,18,19]:

- The specific context (i.e., air traffic and the work environment) in which the controller operates;
- Fully variable personal factors, such as experience, motivation, age, reasoning strategies, attitude, character;
- Physical preconditions (e.g., health, tiredness, and mood).

Therefore, some of the issues that affect the ATC workload are [20]:

- Number of decisions to be taken per unit of time;
- Number of possible strategies for prioritization;
- Adequacy of information in terms of signals perceptibility and data accuracy;
- Consistency between data, command, and control systems;
- Need to perform simultaneously multiple actions;
- System delays in air traffic information or feedback;
- Amount of information to keep in the short-term memory;
- Amount of information to retrieve from long-term memory;
- Seriousness of the consequences of wrong decisions;
- Level of tolerance to system error;
- Physical comfort offered by the ATC working position;
- Working environment (e.g., management of shifts and overtime, relationships with colleagues and superiors).

In the literature, one of the most important studies on ATC workload has been done by Eurocontrol [21]; it is based on the ATC Capacity Analyser (CAPAN) method. CAPAN uses an airspace model in which workload is generated for the simulated working position with given air traffic; it deals with workload of sector air traffic controllers in ACC (Area Control Centre) [22] and not with tower air traffic controllers. In recent years, many studies have investigated the ACC workload [23,24,25,26,27], but understanding of the TATC has not yet deepened.

In this context, Eurocontrol identified the overload threshold of ATC (i.e., the theoretical sector capacity) [21]. It is attained when the time spent by ATC in predefined tasks reaches 70% of the absolute working time (i.e., 42 min during 1 h), and the remaining 30% of the absolute working load is spent for tasks that are not defined within the model and for general recovery (Table 1).

Therefore, a competent airport manager should carry out this analysis on the ATC workload and evaluate the opportunity of upgrade the number of available ATCs before deciding to change the airport infrastructure or/and procedures. The paper presents an innovative methodology to assess the airport capacity with regard to the TATC workload calculated from their radio frequency occupation. Finally, fast time simulation models are used to compare the capacity of the entire airside of an international three-runway airport and its capacity related to TATC: it allows identification of the bottleneck and proposal of countermeasures (i.e., building a new taxiway).

## 2. Materials and Methods

The study is developed through a simulation process to calculate the flight profiles, identify the penetrated airport areas or sectors, and calculate the number of tasks relating to the aircraft that have passed through. The software AirTOp (Air Traffic Optimization, Transoft Solutions Aviation, Gothenburg, Sweden) has been used; it is a fast-time simulation (FTS) platform useful to simulate take-off, route and landing operations, and study airport ground movements [28]. The simulation model is a simplified and virtual replica of a real system; given required levels of precision and detail, it reflects the set of relevant geometrical and procedural characteristics. The algorithms in the platform allow realistic simulations by creating airport models and air spaces in a 3D environment that changes over time and can be configured by setting a series of input variables. The analyses are faster than reality; depending on the number of input data, the software can take a few seconds or minutes to simulate one day of real time.

The CAPAN method has been adapted to evaluate the workload of tower controllers that have different tasks respect to ACTs of ACC. However, two hypotheses of CAPAN have been maintained to define the model for TATCs:

- Time spent on the tasks predefined in the model does not include both surveillance of the general traffic radar, nor are recuperation times recorded;
- The overload working threshold is set at 70% of the absolute working time.

Moreover, the following hypotheses have been added:

- Duration of tasks derives from direct measurement of TATCs listening and speaking time and through interviews with them to quantify the monitoring time;
- Time spent on the predefined tasks does not include the runway and taxiway surveillance from the control tower because visual control of runways and radar are not quantifiable and are assumed to be perpetual throughout the shift;
- Time spent on the tasks does not include the non-frequency coordination (by voice or telephone) between TATCs because both the internal voice coordination between controllers and the telephone use are sporadic and therefore negligible in the proposed model;
- Meteorological conditions are ideal, in absence of extraordinary events [29].

**Figure 1:**
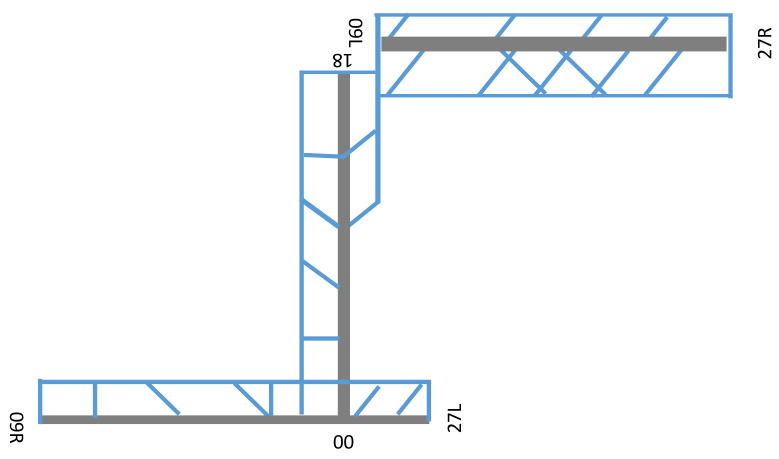
Airport Runways system.

The workload is measured as the frequency occupation time by TATC, whether he is listening or speaking. At this purpose, voice recordings between tower controllers and pilots were taken. Ground controllers (i.e., they deal with ground movement) and air traffic controllers have been interviewed. Their workload includes the problem perception, the reasoning and processing of information to be recorded to the pilot. According to the interviewed controllers, a standard duration time was applied to each task of each workstation; 2 s should be added to each task with speaking and listening time. Furthermore, under hub-in and hub-out conditions (i.e., number of arrivals more than departures, or vice versa, respectively) some configurations may burden the TATC workload. In the FTS model were implemented the task types and obtained durations, the planned airplanes and their flight plans, the aircraft separation procedures (holdings and vectoring areas), the geometrical and functional layout of the runways, the departure and arrival procedures (e.g., standard instrument arrival and standard instrumental departure), and the number and type of air traffic controllers [30]. All input data were consistent with real information from the Aeronautical Information Publication (AIP) of the examined airport, whose layout is composed a complex three-runway system, with two dependent perpendicular runways (i.e., RWY 00/18 and RWY 09R/27L) and the third one independent from the others (i.e., RWY 09L/27R) (Figure 1).

During standard weather conditions (Table 2), RWY 09L/27R (hereinafter L RWY) is reserved to landings, RWY 09R/27L (hereinafter R RWY) is for landings and take-offs, and RWY 00/18 (hereinafter Y RWY) is for take-offs.

The software chooses the landing runway (i.e., L RWY or R RWY). The FTS analyzed the current traffic of the average day of the busiest month (i.e., July), whose average daily movements were 997. Statistical anomalies of traffic volume in the busiest month (e.g., days with unfavourable weather conditions, closure of airside infrastructure, irregular occurrences, and incorrect operation of Air Traffic Management) were ignored in order to identify the representative value of daily movements. It is the value of the occurred daily movements nearest to the statistical average value (i.e., 998). For each of those movements, simulations considered origin/destination, route, aircraft type, airline, flight number, and arrival/departure time. Finally, the runway capacity analyser in AirTOp was used to increase the traffic volume in the airport and calculate the values of workload and runway capacity. A total of three traffic scenarios have been considered: hub-in (60% of arrivals and 40% of departures), hub-out (60% of departures and 40% of arrivals), and mixed mode (i.e., 50% of arrivals and departures). Under standard conditions, one (or two, depending on the traffic volume) ground traffic controller (GTC), one ATC responsible for L RWY (LATC), and one ATC responsible for crossing R RWY and Y RWY (R-YATC) manage the airport movements. In hub-in and mixed modes, due to the huge amount of arrivals, aircraft separation should be evaluated and modified continuously: in this study, each LATC task is increased of 1 s. In hub-out mode departures are from R RWY and Y RWY: airplanes departing from R RWY cross Y RWY. Therefore, each R-YATC task is increased of 1 s. Moreover, in hub-out mode each task of GTC is increased of 1 s due to the high number of taxiing airplanes.

For each movement, the AirTOp identified the needed tasks and calculated the overall workload for each involved controller. Accordingly, the TATC capacity was obtained; it is the maximum number of aircraft that one TATC can manage, maintaining a workload lower than his overload. TATC capacity is an operational capacity, because it quantifies the traffic that the airport system can manage within the defined constraints (e.g., ATC workload, safety, delays). For each scenario, the comparison between TATC capacity and airside airport capacity allowed identification of the system bottleneck for different scenarios. Finally, a modification of the airport layout has been considered in order to compare the effectiveness of different approaches in increasing airport capacity.

## 3. Results

Given the available audio recordings, different tasks types and durations were identified for each analysed air traffic controller. Table 3, Table 4, Table 5 and Table 6 list the tasks durations obtained for ground traffic controller (GTC), LATC responsible for arrivals, R-YATC responsible for departures, R-YATC responsible for arrivals, respectively. Each table is composed of four columns: the left one indicates whether the frequency occupancy value (i.e., duration of task) is due to pilot (P) or ATC (C).

From the apron stand position via the appropriate turnoffs and apron taxiways, a GTC instructs pilots on how to reach the taxiway, avoiding conflicts with other aircraft. The overall procedure is described in Table 3.

During procedure for arrivals, TATC receives from ACC ATC the aircraft arrival sequence. Table 4 lists the tasks that compose the procedure having regard to LATC responsible for arrivals.

Table 5 lists the departure tasks of R-Y ATC. R-YATC manages both departures and arrivals because Y RWY and R RWY are crossing. This condition requires the additional task for clearance crossing 00/18 RWY (Table 5).

Table 6 lists the arrival tasks of R-YATC.

**Figure 2:**
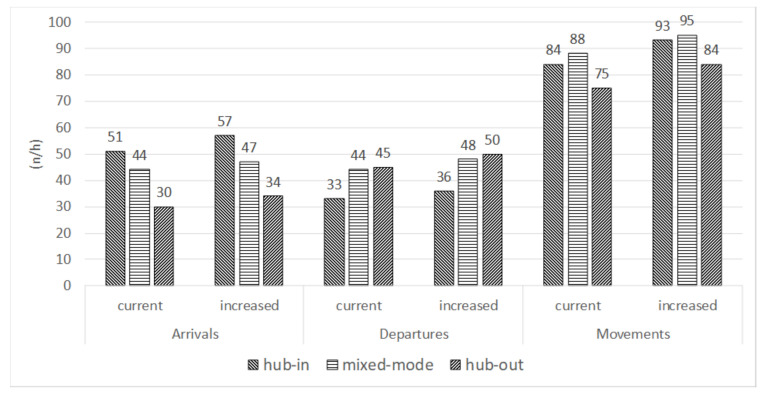
Throughput of each examined scenario.

The frequency occupancy values in Table 3, Table 4, Table 5 and Table 6 allowed calculation of workload in the examined scenarios with increased daily traffic: Figure 2 shows the current and increased throughput, in the most stressed hour (10 a.m.–11 a.m.), for each examine d scenario.

The increased throughput had been considered in order to calculate the ATC workload. Having regard to the hub-in scenario, Table 7 lists the workload during the most-trafficked hours for GTC, LATC, and R-YATC.

**Figure 3:**
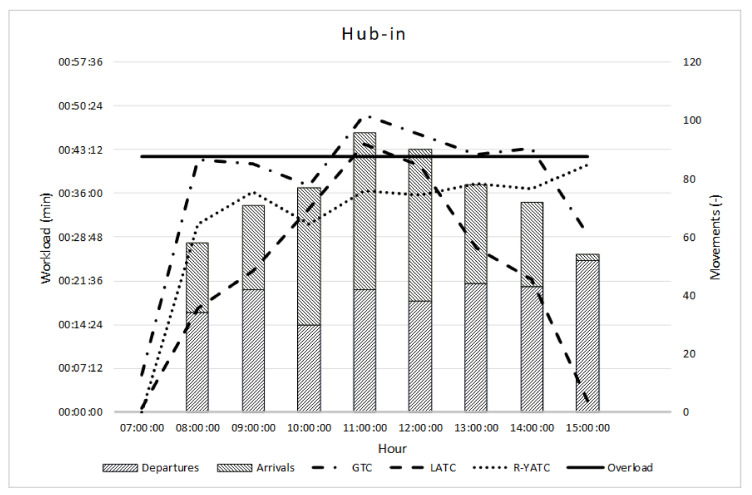
Hub-in workload and throughput.

Figure 3 summarizes the workload e throughput in the hub-in scenario. The bar graph in Figure 3 represents the workload of ATC with different lines (i.e., dash dotted for GTC, dotted for R-YATC, and dashed for LATC); the workload limit (i.e., 42 min) with the continuous line; while the bars represent the number of departures and arrivals (SW–NE and NW–SE lines, respectively).

Under such conditions, the GTC is overloaded for 4 consecutive hours (dash dotted line between 11:00 and 14:00). Moreover, when the arrivals reach the maximum value equal to 54, the LATC has a workload 5% more than the limit (i.e., at 11:00).

**Figure 4:**
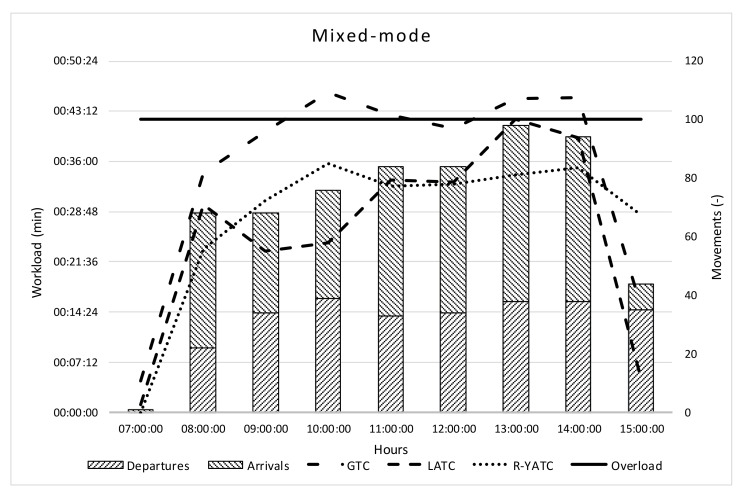
Workload e throughput mixed-mode.

Having regard to the mixed-mode, similar workload conditions are in Figure 4.

In the mixed-mode, the GTC is overloaded for 4 h, and at 13:00 (when 60 arrivals are expected) the overload of LATC is registered.

**Figure 5:**
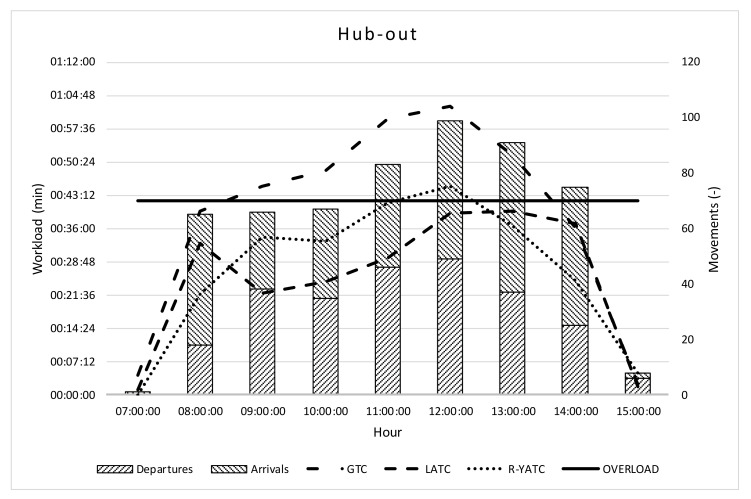
Hub-out workload and throughput.

Figure 5 summarizes the results for the hub-out scenario.

In the hub-out scenario, the GTC is overloaded for 5 consecutive hours (dash dotted line between 09:00 and 15:00); the absolute value of the overload is more serious than in the hub-in scenario (i.e., 62:25 min vs. 48:59 min). This analytical result is not just over the operative capacity of air traffic control, but denotes an impossible condition. The highest number of departures is at 12:00, when the workload of R-YATC is 7% more than the limit (i.e., 45:09 min).

Figure 3, Figure 4 and Figure 5 demonstrate that a single GTC is not enough to manage the traffic within the accepted 10 min-delay. Therefore, a second GTC has been added to the model: GTC1 and GTC2 denote the first and the second ground controllers. Moreover, the taxiway configuration forces all airplanes departing from 18/00 RWY and arriving from 09L/27R RWY to use the same taxiway. In order to solve this bottleneck, the airport management body is studying the opportunity to build a new taxiway.

**Figure 6:**
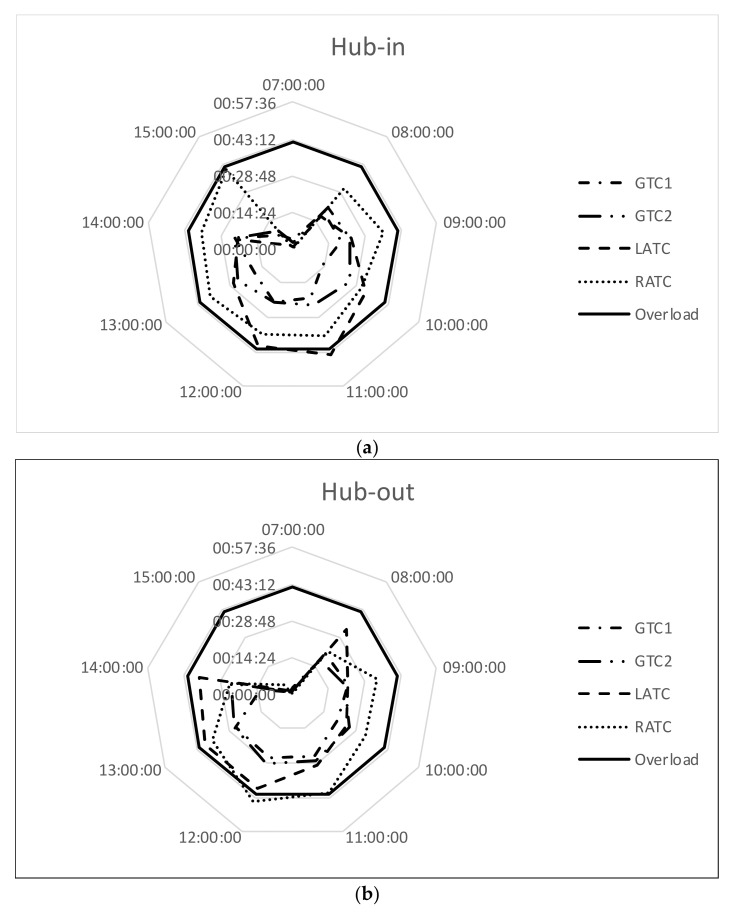

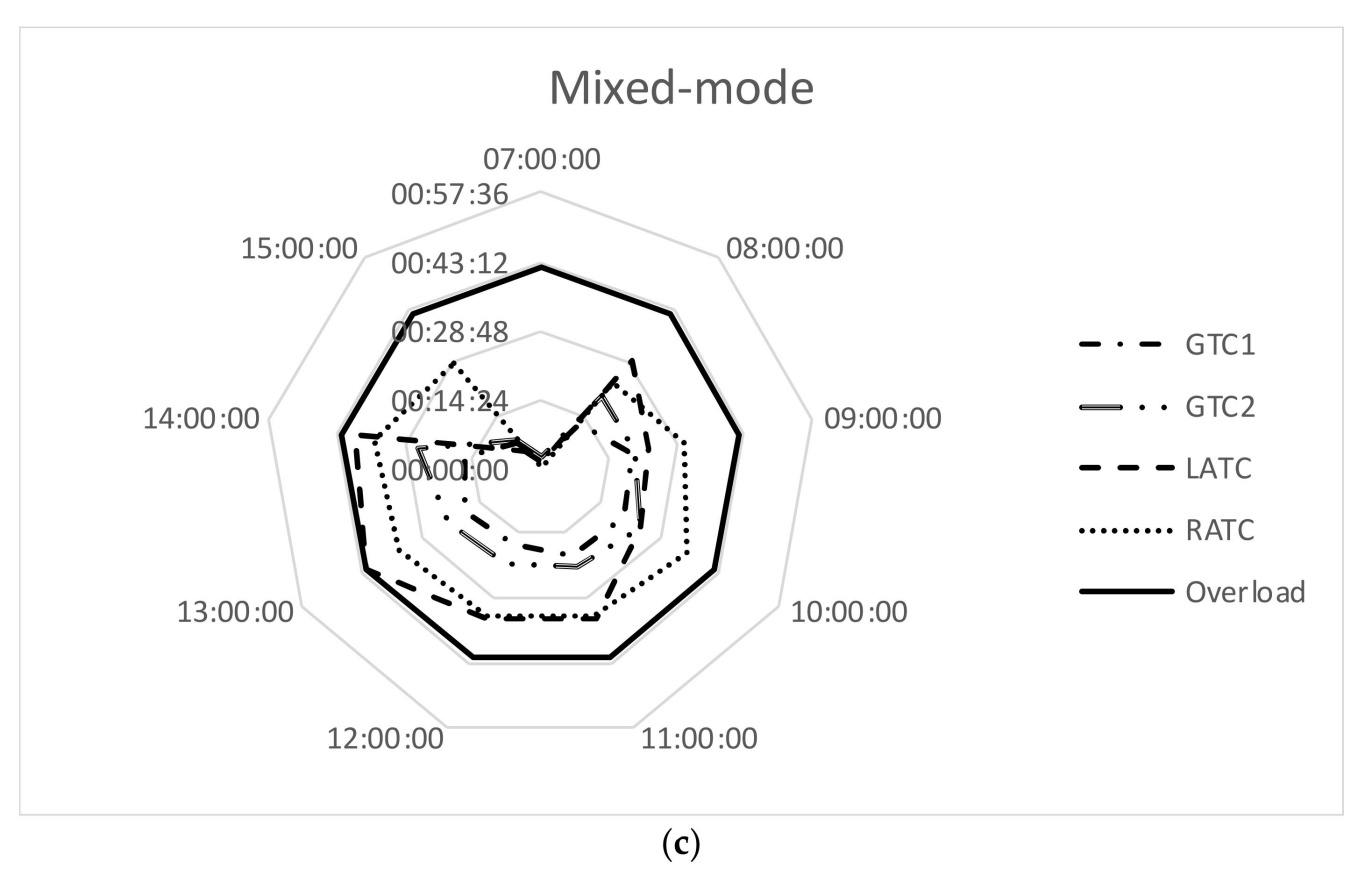
Workload of controllers when 2 GTCs are available: (**a**) hub-in; (**b**) hub-out; (**c**) mixed-mode.

The radargrams in Figure 6a–c represent the workload for hub-in, hub-out, and mixed-modes, respectively; traffic volume and distribution per movement coincides with that investigated in Figure 3, Figure 4 and Figure 5.

Figure 6a–c highlight that at least one ATC has an overload despite the presence of two GTCs: in hub-in, LATC overloads at 11:00; in hub-out R-YATC overloads at 12:00; in mixed-mode LATC overloads at 13:00. Therefore, the second GTC solves the problem of overload for ground controller and the bottleneck becomes the workload of runway controllers.

## 4. Discussion

The results from airside and runway capacity studies revealed:

- GTC manages up to 60 movements/hour under heavy workload conditions (Table 1). For more than 60 movements/hour, two GTCs are necessary;
- R-YATC manages 46 departures/hour under heavy workload conditions:
- L ATC manages 45 arrivals/hour under heavy workload conditions;
- Due to the geometrical configuration of the examined airport, the hub-out mode is the most severe for GTCs because it needs for more tasks than other modes;
- Regardless of the investigated mode and although two GTCs are necessary, the number of movements managed by airside produces an overload condition for at least one runway controller.

**Figure 7:**
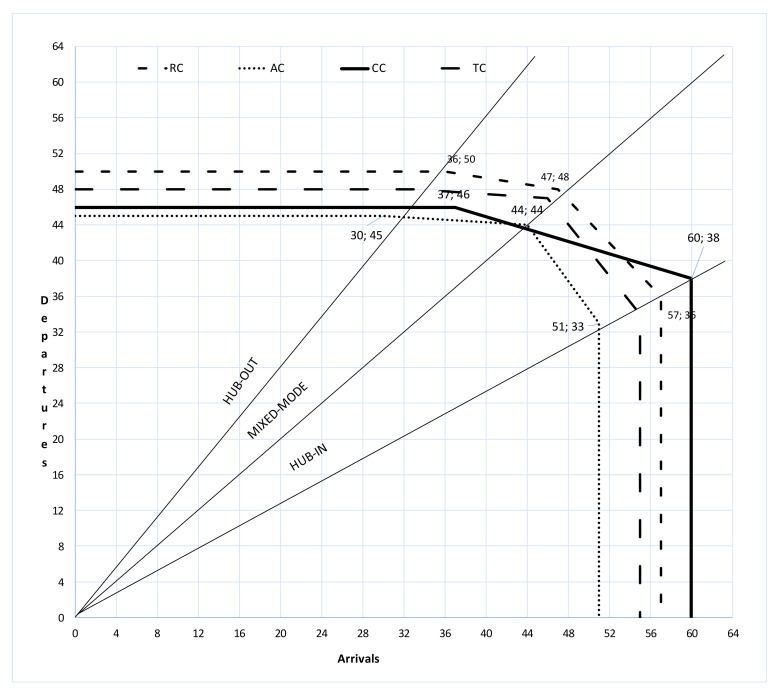
Comparison of runway (RC), current airside (AC), potential airside (TC), and ATC capacity frontiers (CC).

Finally, for each scenario, the authors compared the capacity having regard to the current runways configuration (runway capacity—RC), the current airside capacity (AC), the potential airside capacity (with a new taxiway—TC), and the tower controller capacity (CC). This approach permitted to identify the bottleneck and to evaluate critically possible strategies to improve the system. Figure 7 summarizes the identified Pareto frontiers. They compare the runway, current airside, potential airside, and ATC capacity frontiers at different tradeoffs of arrivals and departures. It is possible to distinguish four borders of hourly throughput.

The runway capacity frontier represents the maximum capacity related to the runway system: this boundary is impossible to be crossed. In the examined airport, the hub-out (i.e., number of departures more than arrivals) configuration gives a lower capacity than the hub-in (i.e., number of arrivals more than departures) capacity because one runway is available for arrivals (Y RWY) and two ones for departures (L RWY and R RWY). Operative hub-out capacity depends on the maximum number of airplanes managed by R-YATC (dashed line in Figure 7) and is given by AC (continuous line in Figure 7). On the other hand, in hub-in and mixed-mode (or balanced mode) the CC frontier is outer than the AC one.

In Figure 7, the AC frontier is the inner curve: whatever the examined mode, the employment of two GTCs is not effective (CC line is outer than AC). Both the procedural (i.e., two working GTCs) and the geometrical (i.e., new taxiway) measures need for enhancing the Pareto frontier. Under such conditions, the operative capacity depends on CC in hub-out and mixed mode and on TC in hub-in: both workload and delays affects the overall airport capacity.

## 5. Conclusions

Given the expected growth in the air transport sector, airport operators aim to increase the number of hourly movements within the limits of safety, admitted delays, and workload of air traffic controllers. In the literature, infrastructural or procedural elements are considered variables that affect the airport capacity, while the human component is often overlooked. However, the tradeoff between capacity expansion and workload of air traffic controllers should be evaluated. In recent years, many studies have investigated the ACC workload, but understanding of the TATC has not yet deepened.

This study aims to evaluate the capacity of air tower controllers defined as the maximum number of aircraft that a tower controller can manage while maintaining a workload lower than the overload (according to Figure 7, the current capacity given by TATCs is (33;46), (44;44), and (60;38) in hub-out, mixed mode, and hub-in configurations, respectively). The overload is a condition when a controller is focused on the tasks predefined in the model not less than 42 min an hour. For different ATCs (i.e., ground and tower air traffic controllers), the occupation times of the radio frequency have been considered to define an innovative and theoretical model. It is based on registered conversations and data from interviews to controllers in order to identify the carried-out tasks. Each task has an execution time, increased to take into account the operational aspects of the examined high-trafficked airport. The FTS software AirTOp has been used to simulate the traffic management in an international three-runway airport. The output data permitted to measure the workload of ATCs and quantify the pertinent operative capacity within the defined overload. The maximum traffic manageable by the infrastructure is generally higher than that manageable by TATCs without falling into the overload condition. The employment of more TACTs could improve the airport operability, but the operative capacity given by the airside cannot be overlooked. In the examined study, TACTs workload limits the airport capacity in hub-out and mixed mode when an improvement in the airport layout (i.e., a new taxiway) is made. On the other hand, the airport capacity in hub-in depends on the runway capacity. Therefore, an airport management company should analyze both procedural and geometrical measures, and their effects in the Pareto frontier compared to the runway capacity, in order to evaluate effectiveness of the adopted strategies.

This study tries to solve the research gap by monitoring the activities carried out by TATCs, listening and speaking their communications, and interviewing them to quantify their monitoring time. The proposed method to calculate the TATC workload could be used in different airports: its modular structure allows modifications and adjustments in order to model the carried-out tasks. When implemented in a FTS software, it provides a tool for management bodies and/or authorities to evaluate strategies, procedures and policies to improve airport capacity.

## Figures and Tables

**Table 1:** Recorded working time during 1 h.

Threshold (%)	Interpretation	Recorded Working Time During 1 h (min)
≥70	Overload	≥42
54–69	Heavy load	32–41
30–53	Medium load	18–31
18–29	Light load	11–17
0–17	Very light load	0–10

**Table 2:** Input data of weather conditions.

Air Temperature (°C)			Humidity (%)	Visibility (km)	Wind Speed (km/h)		Pressure (mb)	Rain (mm)	Gusts
Average	Minimum	Maximum			Average	Maximum			
19	13	23	80	20	11	21	1016	0	absent

**Table 3:** Tasks of GTC.

Person	Task	Duration (s)	Description
P	Request pushback	4	If necessary, P asks for permission to start the pushback procedure; when the stand is self-manoeuvring, P asks for instruction for taxiing.
C	Pushback approval	4	GTC answers to P giving or denying him permission to pushback. In the latter case, C will call P when it will be possible to initiate the pushback.
P	Hear back of pushback approval	3	
P	Request for taxi instructions	3	P asks for instructions for taxiing.
C	Detailed taxi instructions	5	Taxi instruction could be detailed (if GTC needs to check continuously taxi routes) or seamless, according to traffic interactions.
C	set of taxi instructions	8	
P	Hear-back of detailed taxi instructions	4	
P	Hear-back of the set of taxi instructions	6	
C	Frequency change	4	When the airplane arrives to the taxiway, GTC leaves supervision of the airplane to the controller responsible for departures. P is directed by GTC to change frequency from the ground control frequency to the tower frequency.
P	Hear back of frequency change	3	

**Table 4:** Tasks of LATC responsible for arrivals.

Person	Task	Duration (s)	Description
P	Distance from runway information	4	P provides LATC responsible for arrivals his distance from runway.
C	Corrections in descent rate, information about the runway and the sequence	8	LATC responsible for arrivals gives to P necessary corrections in heading and/or descent rate to ensure the aircraft would remain on the runway centreline and glide path to the runway
P	Hear back of corrections and information	4	
C	Clear to land and weather information	6	LATC responsible for arrivals clears to land on RWY and gives weather information
P	Hear-back of landing clearance	4	
C	Procedure to vacate runway and change frequency	4	LATC responsible for arrivals gives to P instructions to vacate runway and change frequency to contact GATC
P	Hear back of procedure	3	

**Table 5:** Departure tasks of R-YATC.

Person	Task	Duration (s)
C	Frequency change	4
P	Hear back of frequency change	3
C	Line up and wait	5
P	Hear back of line up and wait	4
C	Weather information and clear for take-off	5
P	Hear back of clearance for take-off	3
C	Clear for crossing 00/18 RWY	6
P	Hear back of clearance crossing 00/18 RWY	4

**Table 6:** Arrival tasks of R-YATC.

Person	Task	Duration (s)	Description
P	Distance from runway information	4	
C	Corrections in descent rate, information about the runway and the sequence	8	R-YATC gives to P necessary corrections in heading and/or descent rate to ensure the aircraft would remain on the runway centreline and glide path to the runway
P	Hear back of corrections and information	4	
C	Clear to land and weather information	6	
P	Hear-back of landing clearance	4	
C	Procedure to vacate runway and change frequency	4	
P	Hear back of procedure	3	

**Table 7:** Hub-in workload.

Hour	Workload (h:min:sec)		
	GTC	LATC	R-YATC
07:00:00–08:00:00	00:06:06	00:00:41	00:00:00
08:00:00–09:00:00	00:41:30	00:17:06	00:30:57
09:00:00–10:00:00	00:40:50	00:23:11	00:36:14
10:00:00–11:00:00	00:37:12	00:33:31	00:30:57
11:00:00–12:00:00	00:48:59	00:44:06	00:36:25
12:00:00–13:00:00	00:45:43	00:40:30	00:35:39
13:00:00–14:00:00	00:42:27	00:27:00	00:37:36
14:00:00–15:00:00	00:43:23	00:21:49	00:36:49
15:00:00–16:00:00	00:29:11	00:01:48	00:40:44

## Data Availability

Data available on request due to restrictions eg privacy or ethical.
